# The ex vivo human translaminar autonomous system to study spaceflight associated neuro-ocular syndrome pathogenesis

**DOI:** 10.1038/s41526-022-00232-5

**Published:** 2022-10-28

**Authors:** Michael Peng, Stacy M. Curry, Yang Liu, Husain Lohawala, Gaurav Sharma, Tasneem P. Sharma

**Affiliations:** 1 Eugene and Marilyn Glick Eye Institute, Department of Ophthalmology, Indiana University School of Medicine, Indianapolis, IN 46202 USA; 2 North Texas Eye Research Institute, Department of Pharmacology and Neuroscience, University of North Texas Health Science Center, Fort Worth, TX 76107 USA; 3 Mechanical Engineer Consultant, Sunnyvale, CA 94086 USA; 4 Software Engineer Consultant, Indianapolis, IN 46074 USA; 5 Pharmacology and Toxicology, Indiana University School of Medicine, Indianapolis, IN 46202 USA; 6 Stark Neurosciences Research Institute, Indianapolis, IN 46202 USA

**Keywords:** Vision disorders, Neurodegenerative diseases, Neuroscience

## Abstract

Spaceflight-Associated Neuro-ocular Syndrome (SANS) is a significant unexplained adverse reaction to long-duration spaceflight. We employ an ex vivo translaminar autonomous system (TAS) to recreate a human ocular ground-based spaceflight analogue model to study SANS pathogenesis. To recapitulate the human SANS conditions, human ocular posterior segments are cultured in the TAS model for 14 days. Translaminar pressure differentials are generated by simulating various flow rates within intracranial pressure (ICP) and intraocular (IOP) chambers to maintain hydrostatic pressures of ICP: IOP (12:16, 15:16, 12:21, 21:16 mmHg). In addition, optic nerves are mechanically kinked by 6- and 10-degree tilt inserts for the ICP: IOP;15:16 mmHg pressure paradigm. The TAS model successfully maintains various pressure differentials for all experimental groups over 14 days. Post culture, we determine inflammatory and extracellular component expression changes within posterior segments. To further characterize the SANS pathogenesis, axonal transport capacity, optic nerve degeneration and retinal functional are measured. Identifiable pathogenic alterations are observed in posterior segments by morphologic, apoptotic, and inflammatory changes including transport and functional deficits under various simulated SANS conditions. Here we report our TAS model provides a unique preclinical application system to mimic SANS pathology and a viable therapeutic testing device for countermeasures.

## Introduction

Humans undergoing space flight endure significant pathologic consequences^1,2^. Post-flight data of around 300 astronauts has shown that 29% and 60% of astronauts on short-duration and long-duration missions, respectively, experience decreased distance and near visual acuity changes^3,4^.

In 2011, a study conducted by NASA found that astronauts who underwent long duration spaceflight (LDSF) presented with a range of ocular findings including globe flattening, optic disc swelling, choroidal folding, optic nerve sheath swelling and distension, and visual field defects^1,3^. Several of these ophthalmic changes are characteristic of those seen in individuals with increased intracranial pressure (ICP)^5^. Early studies have documented via lumbar puncture, chronic, mild elevation in ICP post-flight among astronauts^6^. Although increased ICP has yet to be directly measured midflight, numerous ground-based analogues of microgravity have suggested that ICP is likely mildly elevated in space^5^. Reports of ground-based studies simulating microgravity for variable positions of head-down tilt (HDT) have demonstrated immediate ICP rise in humans, non-human primates, and other animal models^5,7–10^. As a result, the association of vision changes and increased ICP initially led NASA to refer to the syndrome as the Visual Impairment/Intracranial Pressure syndrome, or “VIIP”^11^. Only later in 2017 has the broader term, spaceflight associated neuro-ocular syndrome (SANS)^3^, been adopted to encompass the wide range of neuro-ocular findings seen in astronauts. It remains one of the most significant health risks in LDSF.

There are several theories how elevated ICP could occur during spaceflight. One hypothesis is that microgravity induces a decrease in cerebrospinal fluid (CSF) drainage. Head ward microgravity-induced redistribution of CSF volume causes CSF to accumulate in the orbital CSF space and prevents a change in direction of flow from the subarachnoid space^12^. Another hypothesis suggests that the orbital optic nerve lymphatic drainage systems could potentially be affected by microgravity-induced cephalad fluid shifts^3^. The venous system and cerebral venous congestion through ventricular enlargement with no parenchymal atrophy, brain upward displacement with narrowing of the subarachnoid space at the vertex, and alterations in water diffusivity could all result in an increase in ICP^6,13^. These changes correlate with LDSF, can persist for several months post flight and substantiate the possibility that altered CSF homeostasis is associated with spaceflight^13^. Another possibility is that elevated ICP causes mechanical compression through compartmentalization of fluid around the nerve between the sheath and nerve that produces axoplasmic flow stasis^14^. An alternate compartmentalization theory is that during LDSF, the optic nerve and globe could be retracted posteriorly, compressing the CSF within the nerve, and causing local elevated pressure dynamics and expansion^15^.

This increased ICP may explain the pathologic changes observed in SANS such as optic nerve sheath expansion, axoplasmic flow stasis, and globe flattening^6^. However, flight crew do not present with other typical signs of elevated ICP such as bilateral papilledema and headaches which has prompted further investigation of the potential role of ICP in pathogenesis of SANS^6^. Further, all astronauts experience the headward fluid shift associated with chronic weightlessness but only approximately 15% of astronauts develop Frisèn grade optic disc edema based on fundoscopic imaging^16^.

Although evidence exists for increased ICP post spaceflight^6^, the mechanisms of damage remain ambiguous. Recent studies have shown that the relationship between ICP and intraocular pressure (IOP) is critical in the pathogenesis of optic nerve diseases^17,18^. Previously, a canine model demonstrated that by controlling IOP and CSF pressure changes, there can be large displacements of the optic disc^19^. Elevating CSF pressure in porcine eyes has also shown increased principal strain within the lamina cribrosa region and retrolaminar neural tissue^20^. This increased strain on the retinal ganglion cells (RGCs) within the lamina cribosa region can contribute to axonal transport blockage and loss of RGCs^20^. A recent study on astronauts also suggested an anterior shift due to a pressure differential between ICP and IOP may contribute to SANS as indicated by optic nerve lengthening and anterior movement of the optic nerve head (ONH)^21^.

Additionally, ICP typically follows a diurnal pattern on Earth as it fluctuates with positional changes^22^. Thus, it has been suggested that the absence of diurnal variations under conditions of microgravity causes the ICP to be mildly elevated at pressures between upright and supine pressures on Earth^17^. Under conditions of microgravity, the mild increase in ICP due to lack of diurnal variations could lead to lower translaminar pressure gradients at the posterior end of the eye. The long-term lower gradient exposure would subsequently lead to optic nerve head (ONH) remodeling and result in the subsequent pathologic changes observed in SANS.

To distinguish the effects of elevated ICP due to shear stress of fluid pressure surrounding the optic nerve, a human model is needed to target disease etiology ex vivo using IOP/ICP. The aim was to isolate the effects of fluid pressure around the ON and kinking of the ON to mimic the in vivo paradigm of SANS. This allowed the ability to distinguish the specific degenerative effects of elevated fluid pressure versus other factors such as sheath elasticity and vascular venous pulsations on the ON. The goal for this study was to understand if various pressure conditions experienced under microgravity were enough to cause pathogenic effects on axoplasmic stasis, ONH remodeling, and other changes to the retina without the complexities of vascular pressure, lymphatic congestion, and sheath compression.

Characterizing a ground-based analog using an ex vivo human model that can mimic aspects of SANS pathogenesis is crucial to not only identify pathways of pathogenesis, but also new therapies to overcome visual neurodegeneration. We have successfully designed a translaminar autonomous system (TAS) that can independently regulate IOP and ICP as well as kinking of the ON to recreate the various conditions under microgravity. We designed a two-chamber system such that the environment surrounding the posterior segment of the human donor eye can be isolated from its ON. Each chamber can be variably pressurized, and the direction of the ON can be changed to mimic aspects of tortuosity observed during space flight. These posterior segments have previously been successfully cultured for 14 days and have shown viability and structural integrity^23^. We hypothesize that our ex vivo human model will provide a system to study SANS pathogenesis and give us a deeper understanding of the relationship between ICP/IOP and ON biomechanical changes.

## Results

### The translaminar autonomous system maintained human donor posterior eye cups for 14 days

**Fig. 1:**
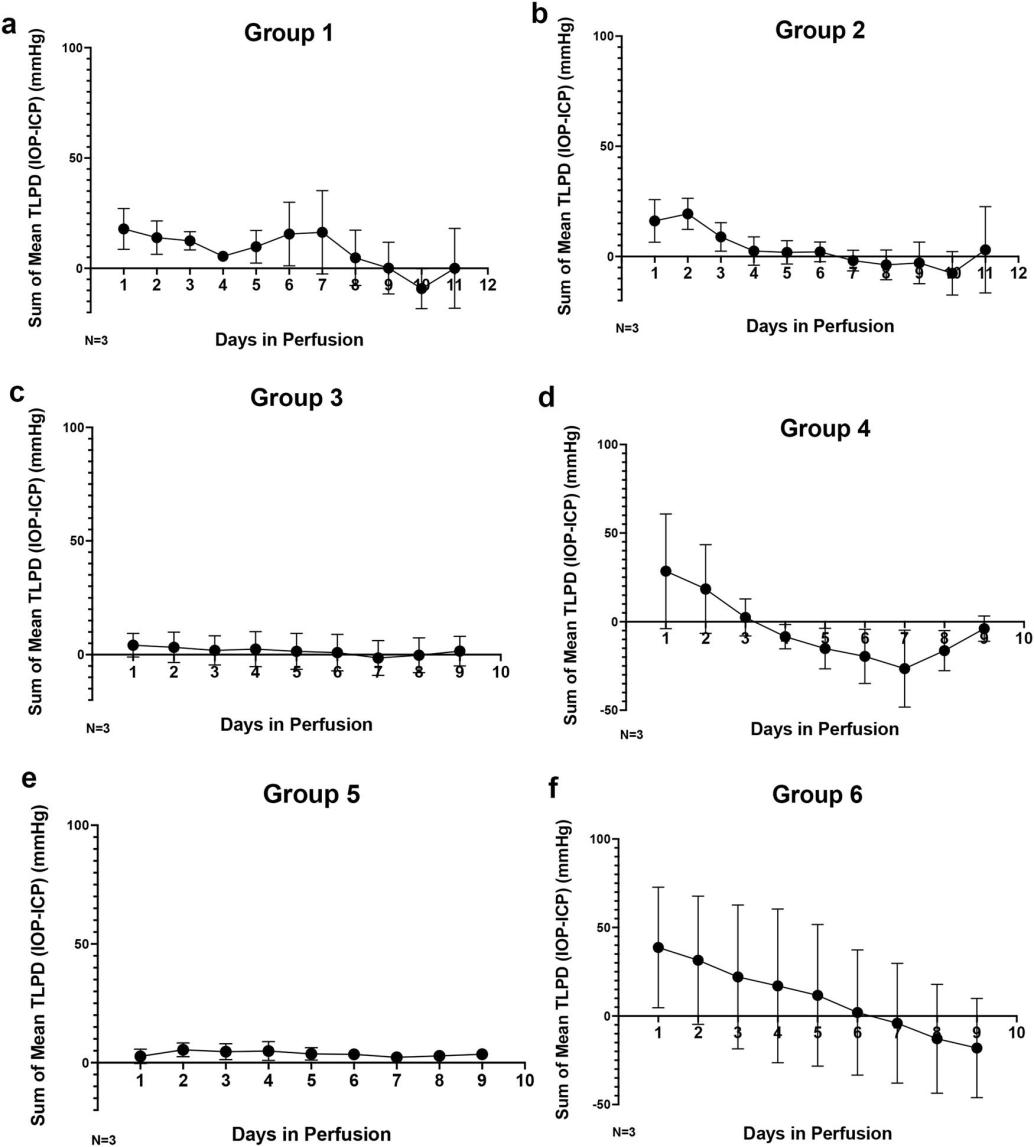
Maintenance of translaminar pressure difference. Graphical representation of sum of mean TLPD (difference in mmHg of IOP-ICP every 24 h) pressures being maintained for (**a**) Group 1, (**b**) Group 2, (**c**) Group 3, (**d**) Group 4, (**e**) Group 5 and (**f**) Group 6. ICP intracranial pressure, IOP intraocular pressure, TLPD translaminar pressure difference, *N* = 3. Data are presented as mean ± standard error of the mean and *P* < 0.05 was considered statistically significant.

The pressurized TAS model (Supplementary Fig. 1) successfully maintained human donor posterior eye cups for 14 days (Fig. 1) (*N* = 3 eyes/group). This model has been previously described in detail regarding the successful individual maintenance of simulated ICP and IOP chamber pressure modulations (Supplementary Fig. 1a)^23^. Here, the model was adapted to simulate ICP and IOP under microgravity conditions as well as the tortuosity of the ON observed after LDSF (Supplementary Fig. 1b)^24^. Representative images (Supplementary Fig. 1c, d) depicting the actual 3D printed models and a representative human posterior eye segment being placed within the TAS model is shown. To improve the accuracy of the pressure settings, we built individualized pumps for each ICP and IOP chamber. This allowed us to modulate the pressures to a 1 mmHg degree of accuracy by manually adjusting the flow rate of our perfusion medium. Some groups required several days before reaching a stable pressure, but pressures were relatively consistent once equilibrium was reached.

The various pressure conditions were determined from previously published data^5,7–10^. On Earth, ICP is lower when seated in the 90° upright posture compared to supine (seated, 4 ± 1 vs. supine, 15 ± 2 mmHg). On the contrary, under conditions of acute zero gravity, ICP is reduced but not to the levels of an upright position on Earth (supine, 17 ± 2 vs. microgravity, 13 ± 2 mmHg). The prolonged exposure to microgravity does not cause a pathogenic elevation in ICP (supine, 15 ± 2 vs. 24 h head‐down tilt, 15 ± 4 mmHg) but instead it prevents the normal lowering of ICP when upright^17^. This chronic, mild elevation in ICP, does not subside under microgravity conditions and can adversely affect eye structure and function during prolonged space flight^22^. In addition to ICP, the IOP is also seen as instantaneously elevated during parabolic flights under acute microgravity exposure. A 5 mmHg increase in IOP is observed through the free-fall phase of parabolic flight from a mean baseline value of 12 mmHg to 19 mmHg within 20 s of exposure to microgravity^25^. It has been shown that during short-duration flights, the IOP initially elevates during the first few days and then drops below preflight levels on return to Earth^26^. Under stimulation studies, IOP during a 6° HDT bed rest study for 7 days has shown an initial immediate rise in IOP after which there has been a slight and progressive decrease by 1.3 mmHg^27^. Variable reports on IOP changes during 6° and 10° HDT in humans have shown variable responses with increase in IOP and ICP through short-term studies^27,28^. Although long-term monitoring of HDT has not been performed in humans.

Based on this data, the following pressure groups were identified: group 1 (12:16 mmHg, ICP: IOP, Fig. 1a), group 2 (15:16 mmHg, ICP: IOP, Fig. 1b), group 3 (12:21 mmHg for 7 days and then 12:16 mmHg for 7 days, ICP: IOP, Fig. 1c), group 4 (21:16 mmHg, ICP: IOP, Fig. 1d), group 5 (15:16 mmHg, 6 degree optic nerve tilt, ICP: IOP, Fig. 1e) and group 6 (15:16 mmHg, 10 degree optic nerve tilt, ICP: IOP, Fig. 1f). We were able to maintain relatively consistent pressures for each of the 6 groups under various ICP: IOP conditions (Fig. 1). We took all average translaminar pressure difference (TLPD) (difference in mean of IOP-ICP) for each group (*N* = 3) and graphed them for each 24-hour timepoint (Fig. 1). Group 3 has been divided into two pressures for the IOP chamber to reflect the acute elevation of IOP during initial flight stages and the subsequent return to normal physiological levels (Fig. 1c). In addition, group 1 (12:16 mmHg, ICP: IOP) (Fig. 1a) reflects the average mean Earth ICP and IOP pressures but at constant pressures.

### Minimal diameter and depth changes pre and post culture of posterior globes amongst all groups

**Fig. 2:**
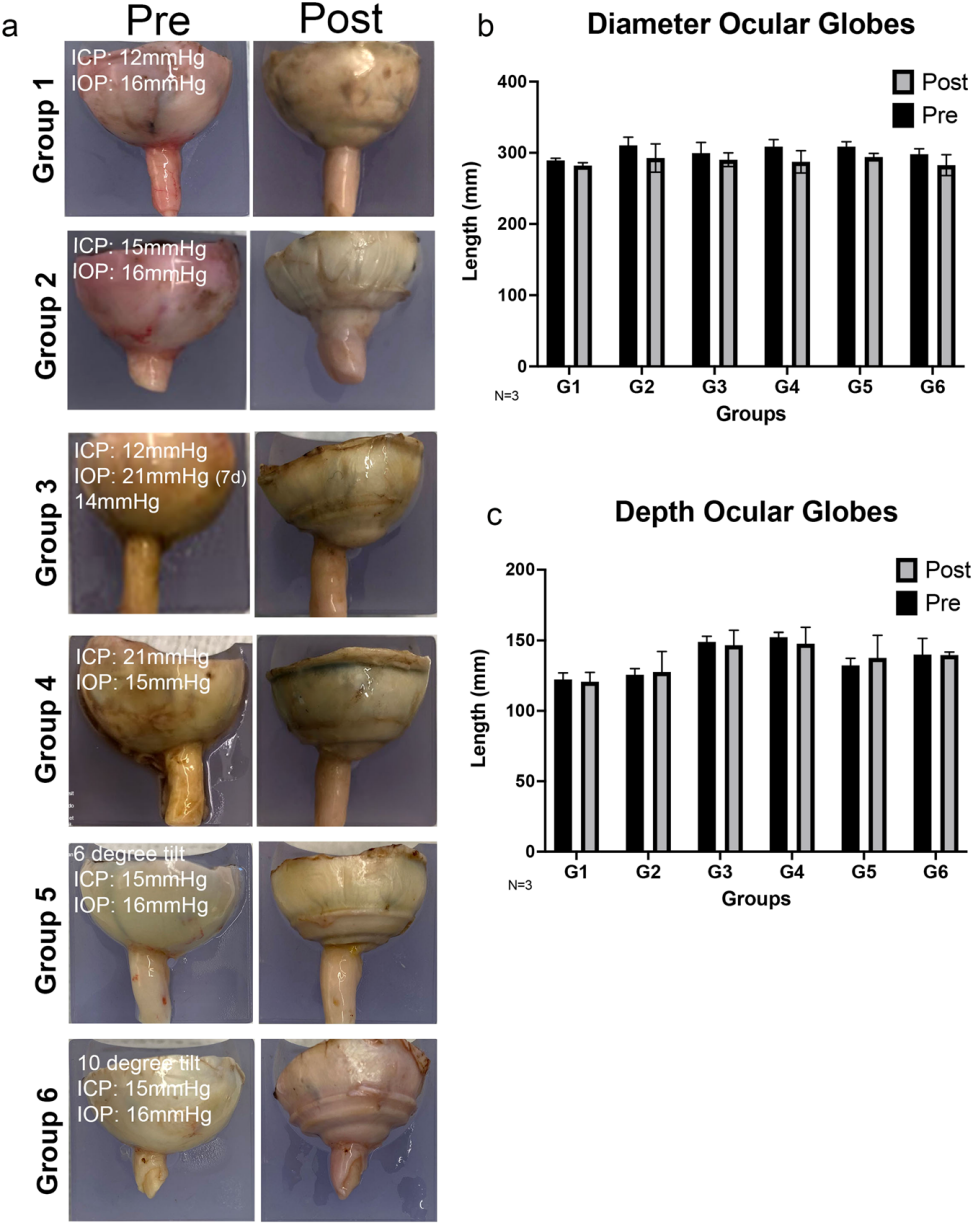
Analysis of posterior human globes pre and post culture in the TAS model. **a** Representative images of posterior globes pre and post 14 days perfusion culture. Graphical representation of pre-post values of (**b**) diameter and (**c**) depth of posterior globes (*N* = 3). Length in pixels calculated from Adobe Photoshop. ICP intracranial pressure, IOP intraocular pressure. Data are presented as mean ± standard error of the mean and *P* < 0.05 was considered statistically significant.

Representative images of posterior globes are presented for each group (Fig. 2a). Notably, there were overall minimal gross changes to the posterior eye cups post perfusion culture. Visual depiction of the posterior globes did present some bulging at the ONH. This may be because of swelling within the region due to chronically elevated intracranial pressure or the mechanical strain induced on the tissue due to pressurization within a sealed system (Fig. 2a). However, no significant changes in the diameter or depth of the posterior cups were observed post culture for all groups (Fig. 2b, c).

### Expression of retinal markers with increase in apoptosis and inflammation markers post culture

**Fig. 3:**
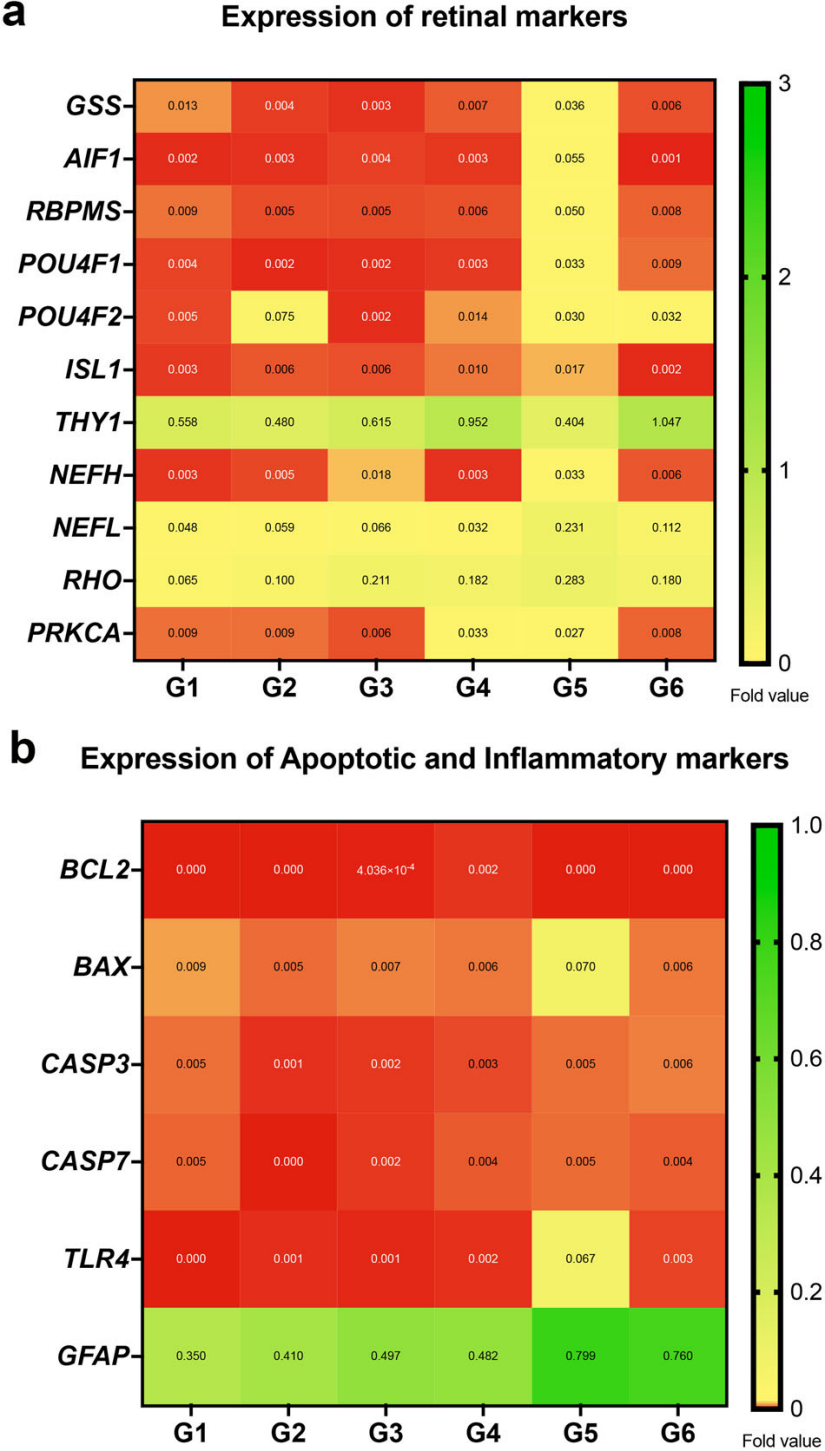
Expression of retinal markers with increase in apoptosis and inflammation in experimental groups post culture. Heatmap of (**a**) retinal markers and (**b**) apoptotic and inflammatory markers in control and experimental groups (*N* = 3). Mean fold values are indicated for each marker. G1 group 1, G2 group 2, G3 group 3, G4 group 4, G5 group 5, G6 group 6.

We analyzed the retinal markers from peripheral retinal tissue samples post TAS perfusion culture from each group (*N* = 3) through TaqMan array analysis. We observed expression of all crucial RGC and retinal markers (Fig. 3a) in each group, demonstrating RGC survivability post culture even after 14 days. We also observed expression of apoptotic and inflammatory markers among all groups (Fig. 3b). Importantly, group 1 did not differ significantly from the other experimental groups, suggesting that even non elevated ICP may demonstrate degenerative consequences under simulated microgravity conditions. While there was no significant difference in biomarker expression levels among all groups, we found that group 5 showed a global increase in RGC and retinal marker expression levels (Fig. 3a) as well as elevated BAX (apoptotic) and TLR4 (inflammatory) expression levels compared to the other groups (Fig. 3b). Higher retinal markers may be because of increased tissue preservation post perfusion culture. The increase in BAX and TLR4 suggests that a slight ON tilt in conjunction with elevated ICP could potentially have a greater degenerative impact. This coincides with groups 5 and 6 also demonstrating the highest expression levels of GFAP compared to groups 1–4 (Fig. 3b).

### Secreted ECM proteins in ICP and IOP chambers

**Fig. 4:**
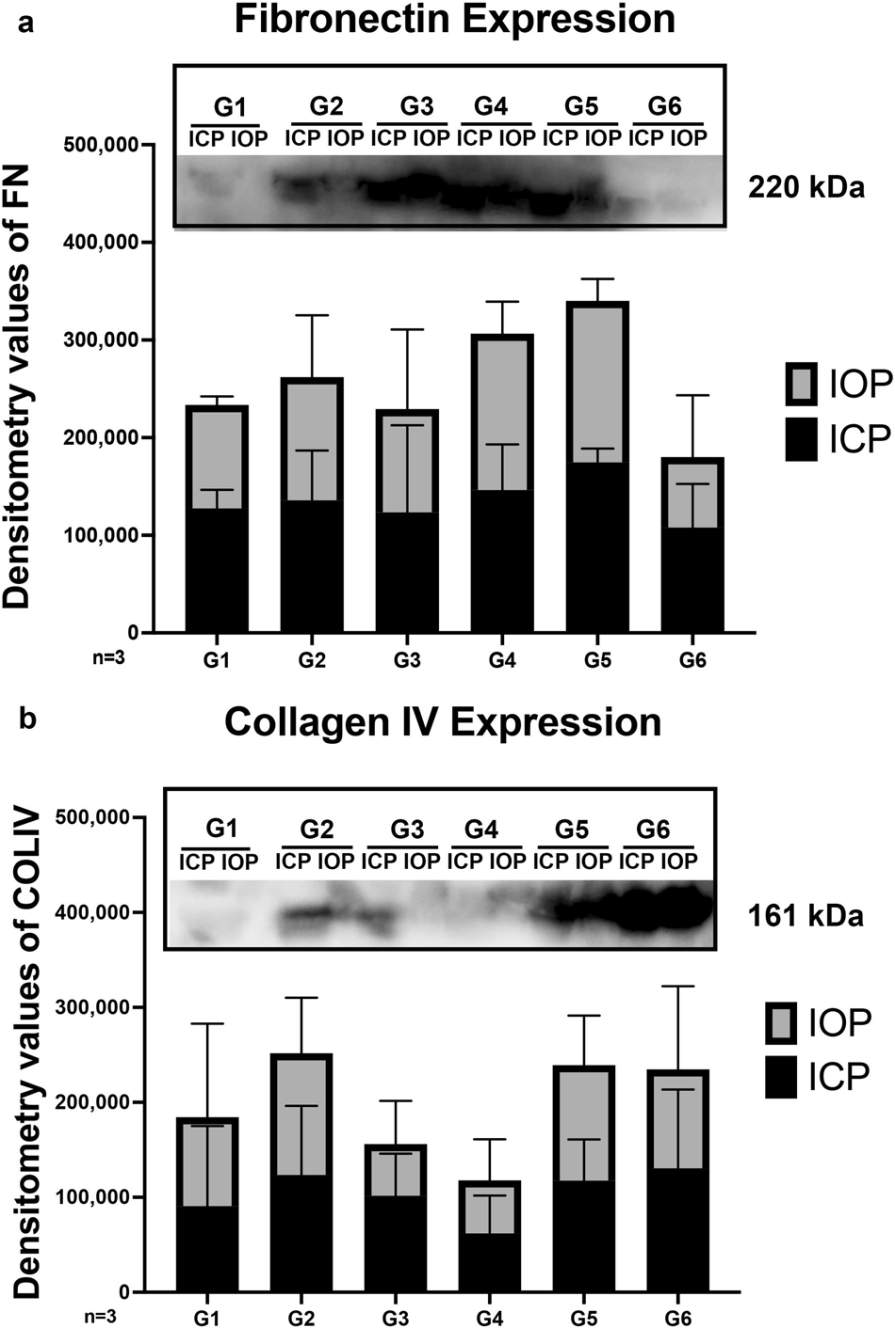
Extracellular matrix secretion observed in ICP and IOP chamber conditioned medium of experimental groups. Conditioned medium collected from the IOP and ICP chambers of every group throughout 14-day perfusion culture. Densitometric values normalized to control after Western blot analysis was evaluated for expression of (**a**) FN and (**b**) COLIV. ICP intracranial pressure, IOP intraocular pressure, G1 group 1, G2 group 2, G3 group 3, G4 group 4, G5 group 5, G6 group 6, *N* = 3. Data are presented as mean ± standard error of the mean and *P* < 0.05 was considered statistically significant.

We were able to measure the secreted extracellular matrix (ECM) proteins from each individual ICP and IOP chamber. We analyzed the expression levels of FN and COLIV collected from the conditioned medium for each group. While we did not observe any significant difference in expression levels across experimental groups, higher levels of FN and COLIV were secreted in the IOP chamber compared to the ICP chambers across all groups (Fig. 4a, b). This suggests increased ECM deposition and secretion is primarily occurring intraocularly at the ONH.

### Increased degeneration of optic nerve axons observed in all groups

**Fig. 5:**
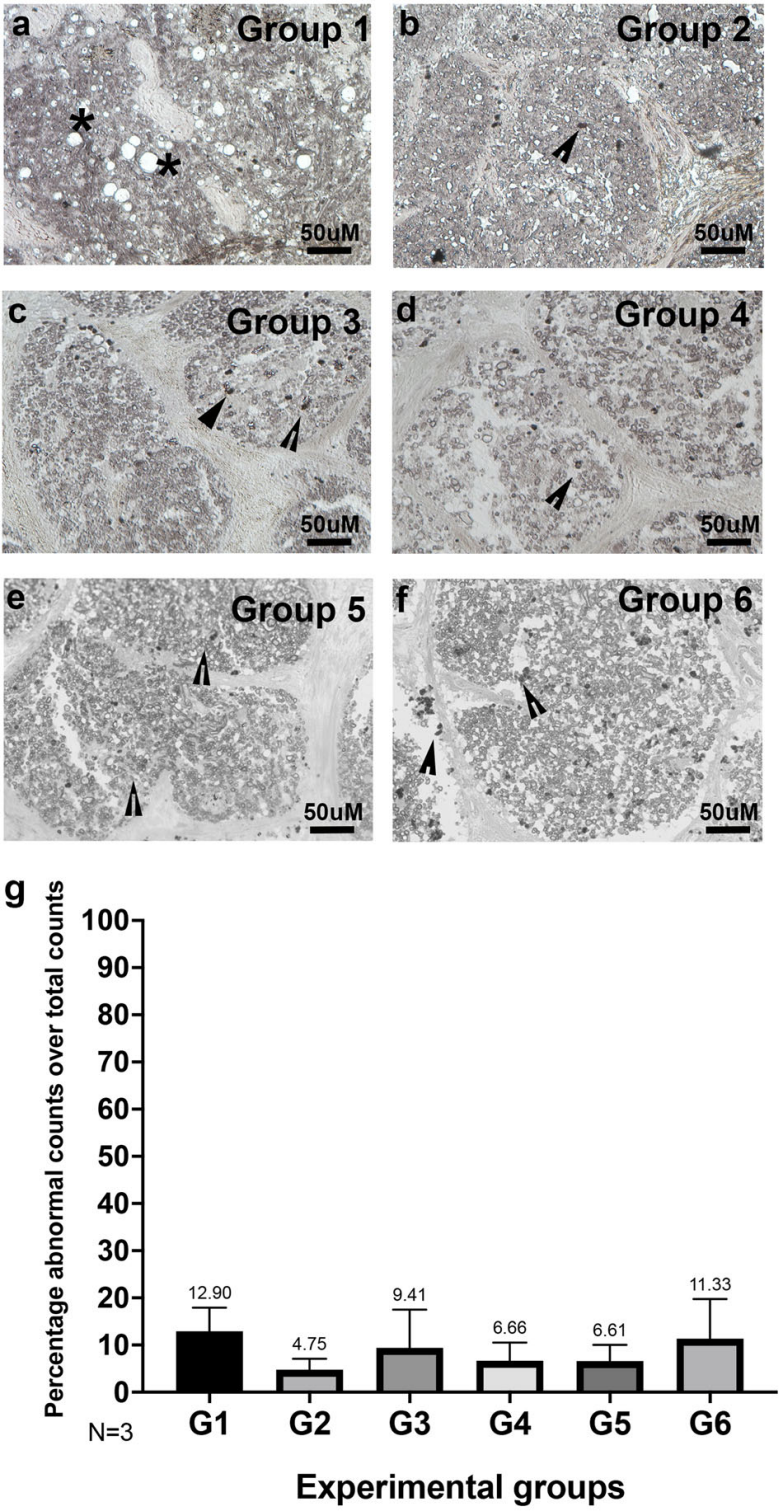
Degeneration of optic nerve axons observed under experimental conditions. Paraphenylenediamine (PPD) staining of the optic nerve was utilized to assess degeneration of axons within the nerves of (**a**) Group 1, (**b**) Group 2, (**c**) Group 3, (**d**) Group 4, (**e**) Group 5 and (**f**) Group 6 cultured for 14 days within the TAS model. Normal and healthy axons (asterix); damaged or dying axons (arrowhead). **a**–**f** 400x magnification. **g** Ratio of abnormal to total axon counts normalized to control, *N* = 3. Data are presented as mean ± standard error of the mean and *P* < 0.05 was considered statistically significant.

Paraphenylenediamine (PPD) staining of the ON was utilized to assess degeneration of axons. PPD lightly stains the myelin sheath of healthy axons while the axoplasm of damaged or dying axons is darkly stained. In moderate to severely affected areas, myelin or cellular debris from degenerating axons are stained black by PPD. Normal and healthy axons shown in group 1 (asterisk) (Fig. 5a) are compared to damaged or dying axons (arrowhead) in other groups (Fig. 5b–f). While there was no significant difference between groups (4.75% to 11.33%, Fig. 5g), group 1 shows the most degenerated axons at 12.9% (Fig. 5g). This suggests that axon degeneration may be seen at constant pressure conditions even at physiologic ICP and IOP.

### Morphologic reorganization with increased expression of extracellular and inflammatory markers within the optic nerve head

**Fig. 6:**
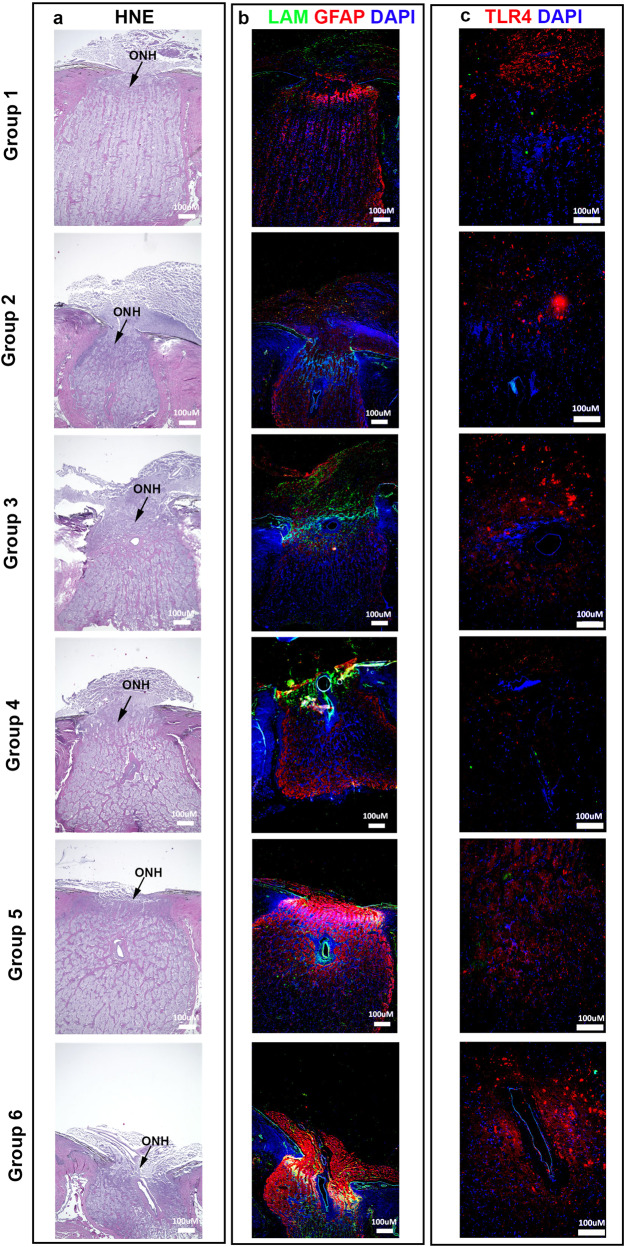
Morphological restructuring of the optic nerve head after perfusion culture. Cross sections of human ONH depict (**a**) H&E staining of Group 1, Group 2, Group 3, Group 4, Group 5 and Group 6. Expression of (**b**) LAM, GFAP with DAPI in the ONH of all 6 groups. Expression of (**c**) TLR4 with DAPI in the ONH of all 6 groups. LAM = green; GFAP, TLR4 = red; DAPI = blue; **a**, **b** 40x magnification; **c** 100x magnification. H&E hematoxylin and eosin stain, ONH optic nerve head.

**Fig. 7:**
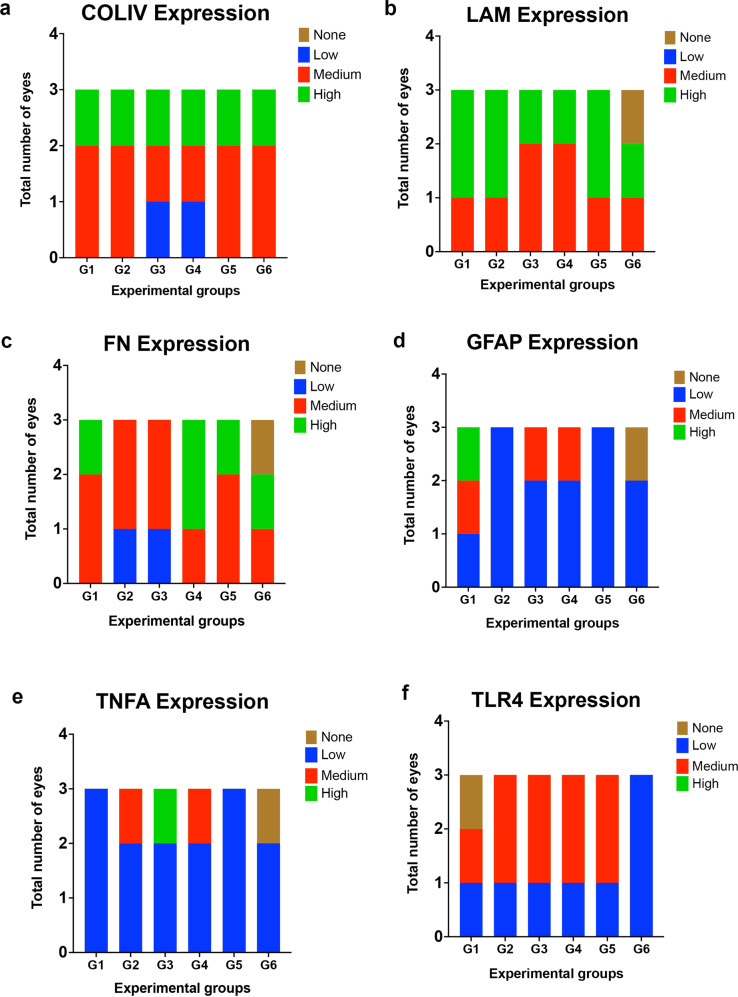
Expression of inflammatory markers at the optic nerve head after perfusion culture. Expression within all groups (**a)** COLIV, (**b)** LAM, (**c)** FN, (**d)** GFAP, (**e)** TNFα and (**f)** TLR4 as graded by blinded observer. Color coding for expression patterns; brown = none, blue = low, red = medium, and green = high, *N* = 3.

We observed increased thickening of laminar beams and restructuring of the ONH (Figs. 6a–c; 7a–f) within all groups. Groups 5 and 6 depicted the slight tilting of the ON as expected. Extensive glial scarring and cupping was also observed in groups 5 and 6. High expression levels of LAM was found within the laminar region of the ONH for all experimental groups (Fig. 6b).

Elevated levels of GFAP among all groups also suggests an increase in inflammation and correlates with the elevated GFAP observed within the retinas (Fig. 3b). Further, elevated levels of the inflammatory marker TLR4 was also observed, specifically in group 6 (Fig. 6c). Degree of expression for all the groups was also analyzed for each of the markers: COLIV (Fig. 7a), LAM (Fig. 7b), FN (Fig. 7c), GFAP (Fig. 7d), TNFα (Fig. 7e) and TLR4 (Fig. 7f). We detected increased COLIV and TLR4 (Fig. 7a, f) expression in all groups with low expression detected for TNFα (Fig. 7e).

### TUNEL positive cells identified within the optic nerve head

**Fig. 8:**
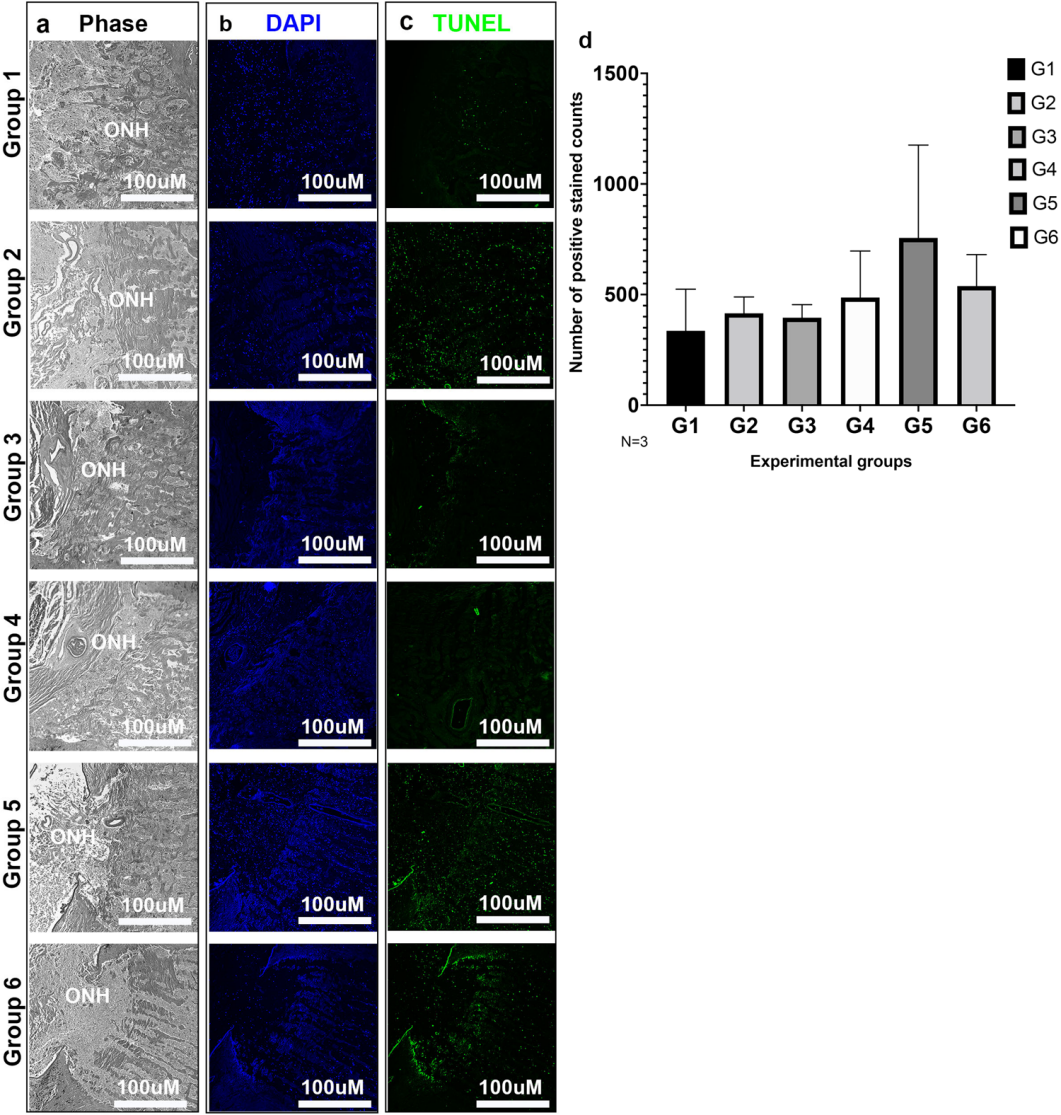
Increased TUNEL positive cells identified within the optic nerve head of experimental groups. Cross sections of human ONH depicting (**a**) phase contrast micrographs of Group 1, Group 2, Group 3, Group 4, Group 5 and Group 6. Expression of (**b**) DAPI in the ONH of all 6 groups. Expression of (**c**) TUNEL positive cells in the ONH of all 6 groups and (**d**) quantification of TUNEL positive cells. DAPI = blue; TUNEL stain = green; **a**–**c** 100x magnification; TAS translaminar autonomous system, ONH optic nerve head, *N* = 3. Data are presented as mean ± standard error of the mean and *P* < 0.05 was considered statistically significant.

High magnification micrographs (Fig. 8a) of the ONH depicted changes in morphology and remodeling of the tissue after perfusion culture under simulated SANS pathogenic changes. To identify apoptosis occurring within the ONH, TUNEL assay was performed (Fig. 8b, c). TUNEL positive cells were shown across all groups with group 5 demonstrating the highest expression levels (Fig. 8d). The remaining groups showed lower expression levels that were similar to those in group 1. However, weak fluorescence does not necessarily indicate a lack of apoptosis as TUNEL positivity only gives a snapshot of the apoptosis occurring continuously. It is possible that extensive cell death may have already occurred but was not detected by staining.

### Expression of CTB within the optic nerve head cross-sections

**Fig. 9:**
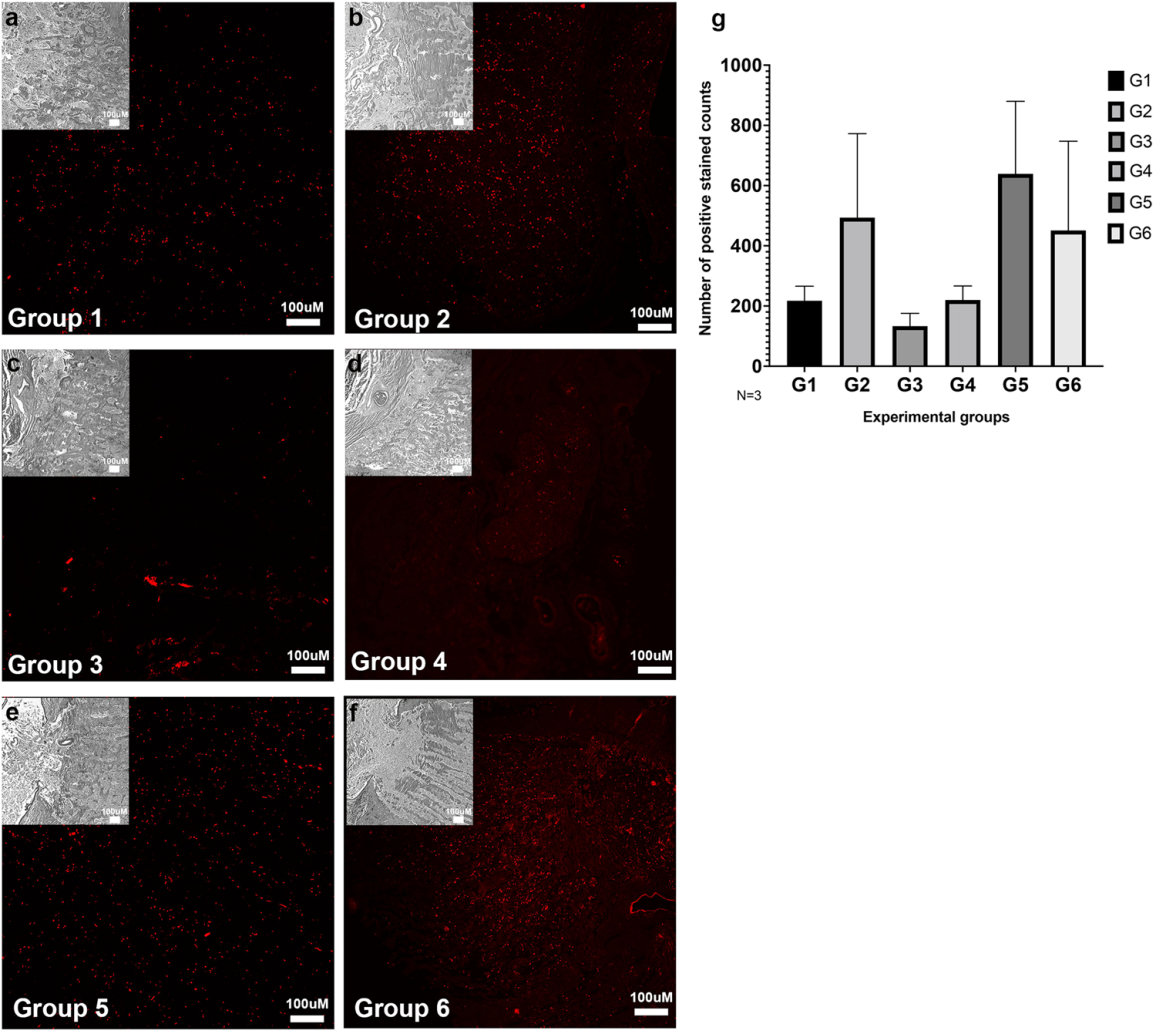
Minimal expression of Cholera Toxin B within the optic nerve head cross-sections. Human ONH depicting phase contrast micrographs (insets) and expression of cholera toxin B (CTB) within (**a**) Group 1, (**b**) Group 2, (**c**) Group 3, (**d**) Group 4, (**e**) Group 5, (**f**) Group 6 cross sections and (**g**) quantification of all CTB positive cells within all 6 groups. **a**–**f** 100x magnification; CTB = red, *N* = 3. Data are presented as mean ± standard error of the mean and *P* < 0.05 was considered statistically significant.

Cholera toxin B (CTB) was perfused through the IOP chamber to measure the retrograde transport of the protein past the ONH (Fig. 9). Expression of CTB was lowest among groups 3 and 4, which had either elevated IOP or ICP conditions, respectively (Fig. 9g). This suggests that a greater TLPD may be an important contributing factor affecting retrograde transport through the ONH. In contrast, higher expression was observed for all other groups (Fig. 9a–f), indicating that average normal IOP and ICP including ON tortuosity did not have as significant an impact on axonal transport.

### Increased retinal dysfunction with high IOP and tortuous ON conditions

**Fig. 10:**
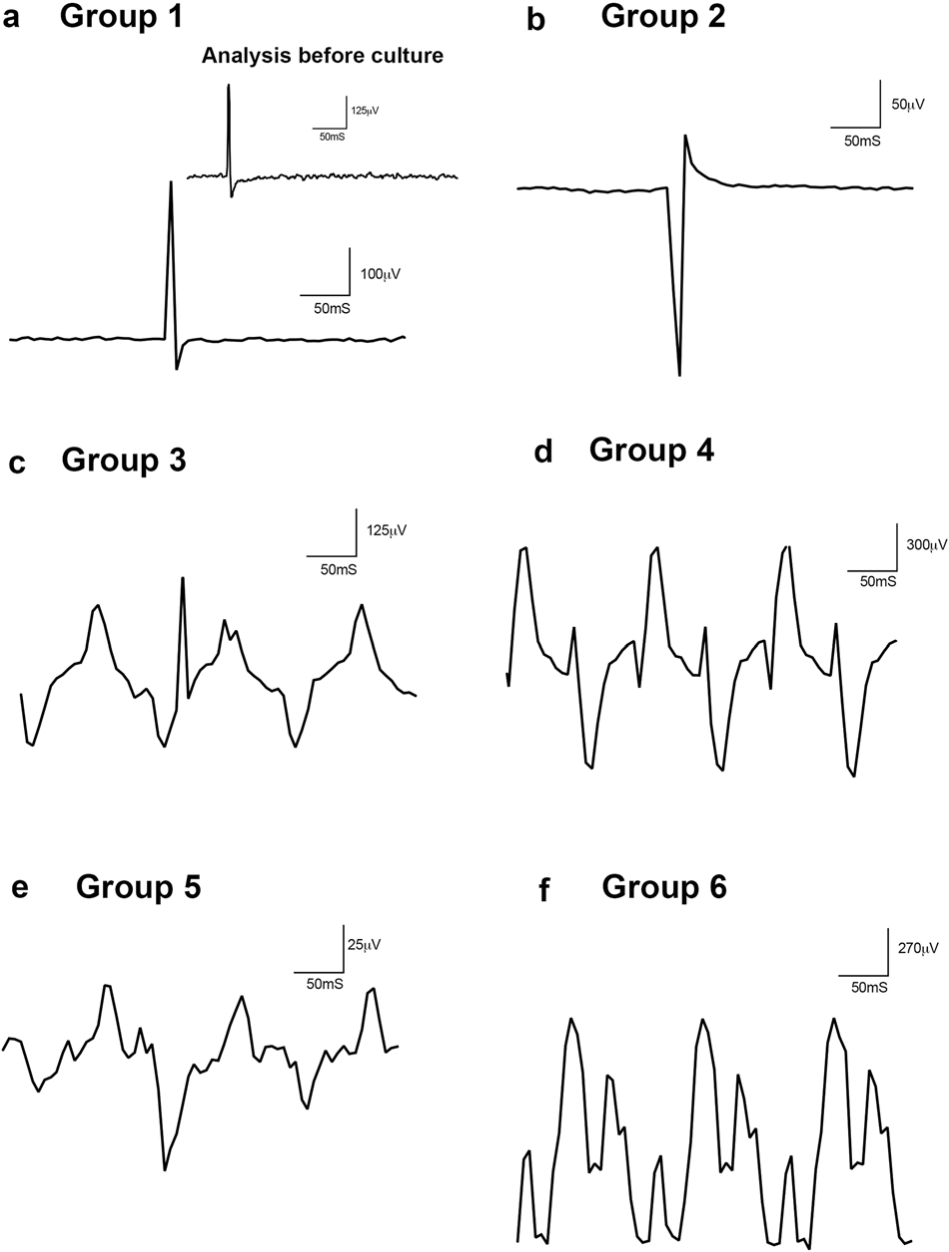
Increased retinal dysfunction with high IOP and tortuous ON conditions. Full field flash ERG recordings on retinas after 14 days perfusion culture. The amplitudes of scotopic threshold responses were measured from the baseline to the positive peak of each waveform and latency measured by time-to-peak major positive deflection for (**a**) Group 1, inset- analysis before culture of a fresh postmortem human retina, (**b**) Group 2, (**c**) Group 3, (**d**) Group 4, (**e**) Group 5 and (**f**) Group 6 from 0.3 mcd.s/m^2^ to 1000 mcd.s/m^2^.

We performed full field fERG recordings on retinas after perfusion culture for 14 days. The amplitudes of scotopic threshold responses were measured from the baseline to the positive peak of each waveform and latency measured by time-to-peak major positive deflection. This response is often referred to as the “mixed rod-cone response,” as there are contributions from both rods and cones to the a-wave. The b-wave is generated by ON- and OFF-type bipolar cells. Compared to groups 1 and 2, representative ERG responses from other groups showed varying amplitudes at 1000 mcd.s/m2 (Fig. 10). Group 1 depicted similar ERGs to the representative functional response for the retina prior to culture, which is displayed as an inset of Fig. 10a. This highlights that group 1 still demonstrates a functionally stable response as compared to a fresh postmortem eye despite showing degenerative changes. Interestingly, groups 3–6 depict waveforms comparable to a rapid flicker sequence illustrating degeneration of the retina under the high IOP and tortuous ON conditions (Fig. 10c–f). This signifies that even though axonal transport may not be affected by ON tortuosity, it may affect the functional capacity of the retinal neurons. Rapid flicker assesses cone-pathway function because rod photoreceptors generally cannot follow rapid flicker.

## Discussion

Using the TAS perfusion culture model, we were able to study the effect of translaminar pressure changes on the eye relevant to SANS microgravity conditions. Our system is an ex vivo human model to study SANS pathogenesis by independently modulating IOP, ICP, and ON tortuosity conditions to simulate aspects of changes observed in SANS. Trends of degeneration were observed among all groups. Surprisingly, the group 1 model system which simulated normal, average Earth IOP and ICP under constant pressure conditions also showed degenerative immunohistochemical changes (Fig. 6) and increased axonal degeneration (Fig. 5). This suggests that diurnal patterns of fluctuation between ICP and IOP which are absent under microgravity conditions may also be conducive for normal Earth pressure dynamics on the ONH. It is possible that the ONH must morphologically change to circumvent the new constant IOP and ICP pressures observed under these environments.

An important consideration for the TAS model was ensuring the viability and functionality of the postmortem human donor eye for the duration of the perfusion culture. This study demonstrates that human postmortem tissue can be a valuable resource for translational research. Human postmortem tissue has been extensively studied^29,30^ and a documented report of 1068 postmortem donor tissues analyzed for RNA quality confirmed that postmortem human brain tissue collected over decades can serve as high quality material for the study of human disorders^31^. In addition, expression profiling of ocular human donor eye tissues postmortem has been performed successfully^32^. Gene expression plier values for apoptosis genes were shown to be minimal or non-existent in this dataset for retinal tissue 6 h postmortem^32^. Therefore, we used the 6-hour time-point as our inclusion criteria for donor eyecup collection. To assess the survivability of the RGCs after the 14-day perfusion culture within the TAS model, we observed expression of crucial RGC and retinal markers for each of the groups. We were also able to successfully acquire ERGs from the retinal tissue. The CTB assay was also able to demonstrate retrograde transport across groups post culture. Collecting conditioned medium allowed the measurement of specific biomarkers that may be utilized in the future to monitor the effects of specific targeted therapeutics perfused through the model system.

After perfusion in the TAS model, we observed degenerative changes associated with increased ECM deposition and inflammation, reflected by the elevated levels of LAM and GFAP as well as elevated levels of secreted FN and COLIV in all groups. RGC axons are acutely sensitive to changes in the lamina cribrosa as they must pass through this network of ECM proteins before converging into the myelinated ON. Thus, pathogenic ECM deposition at the ONH is often characteristic of neurodegenerative diseases due to RGC axon damage. Indeed, PPD staining of the optic nerves demonstrated degenerated axons across all groups. The progressive degeneration of ON axons is a critical pathological event that leads to vision loss. These changes were also reflected in the expression of apoptotic and inflammatory markers observed in the retinal tissues post perfusion culture. The ability to assess these characteristics of neurodegenerative diseases utilizing the TAS model indicates that we can successfully assess the biomechanical alterations that occur with varying ICP and IOP conditions as observed in spaceflight missions.

Notably, group 1 eyes demonstrated several pathological similarities with the other experimental groups. This is evident of a true microgravity condition where the diurnal rhythms of ICP and IOP as observed on Earth are not present. Group 1 also acted as a model for astronauts who may not experience mildly elevated ICP but still develop degenerative changes under constant pressure conditions. Our findings suggest that non elevated ICP at physiologic pressures under microgravity conditions (Group 1) may still yield degenerative changes as observed for the various SANS conditions (Groups 2–6). Further, studies are required to determine if the pathological changes are directly associated with the constant pressure conditions as seen with all our groups. Adjusting the ICP and IOP according to awake and sleeping positions could simulate the natural fluctuation of pressures on Earth. We can perform these diurnal pattern changes using a continuous perfusion system (Supplementary Fig. 3). Future studies aim to mimic Earth’s diurnal pattern changes by regulating pressures through adjustment of flow rates via an automated feedback software application (Supplementary Fig. 3b, d). This would allow us to assess if these degenerative changes could be alleviated by incorporating a diurnal Earth pressure control.

The current model nevertheless allowed us to study the pathogenic paradigm of ICP and IOP without the stressors of other stressor paradigms which are implicated in SANS, such as venous and lymphatic changes^1,6^. Current hypothesis suggests that the mismatch between IOP and ICP may result in reorganization of the ONH seen in SANS^22^. Gravity on Earth influences ICP changes in humans, which is generally lower for most of the day. This could potentially lead to low ICP pressure gradients at the posterior end of the eye, causing a greater TLPD. Long term lower gradient exposure in microgravity due to elevated ICP would subsequently lead to ONH remodeling^33,34^. Another leading alternate hypothesis is the compartmentalization of CSF within the optic nerve and sheath^3,35^. The tightly confined space could potentially generate a detrimental flow equilibrium even without elevated CSF pressure within the brain^36,37^. This pressure dynamic has shown to cause axoplasmic flow stasis within the nerve fiber layer and prelaminar region^14^. Additionally, it has been documented in an experimental chronic intracranial hypertension rhesus monkey model that raised CSF pressure causes optic disc edema. However, opening the optic nerve sheath resolved optic disc edema, demonstrating that elevated CSF pressure alone does not cause pathogenesis. It is instead due to a mechanical phenomenon from the compartmentalization of fluid around the nerve through the sheath that produces axoplasmic flow stasis^14^. This theory corroborates the ocular findings but not the pituitary dome compression found in a subset of astronauts^38^. A different compartmentalization theory is that during LDSF, the optic nerve and globe could be retracted posteriorly, compressing the CSF within the nerve, and causing local elevated pressure dynamics and expansion^15^. With either theory, the increased pressure could possibly be transmitted directly through the subarachnoid space to the posterior sclera resulting in posterior globe flattening, folding of the choroid and axial shortening^3^. Our model would allow us to test the effects of compartmentalization with the optic nerve sheath. Even though in this study we wanted to remove the compartmentalization effects of the elevated ICP and just study the mechanical dynamics of changes happening at the ONH through IOP and ICP changes, future studies could potentially include looking at the compartmentalization aspects by keeping the sheath of the optic nerve intact for experiments.

Further, microgravity fluid shifts during spaceflight has shown to cause jugular venous distension and mild thickening of the retinal nerve fiber layer^39^. This does not specifically corroborate that jugular venous distension causes elevated venous pressure during spaceflight, and there are conflicting debates on the increase or decrease of venous pressure. However, the impact of retinal pressure and role of the glymphatic system also remains a point of debate^40,41^.

Even though the TAS model allows us to characterize multiple degenerative changes, there are several limitations to the model. A caveat to the model is the lack of blood circulation within the cadaveric eye, so the effects of blood pressure cannot be studied. Importantly however, this also allows us to specifically delineate the pathogenic effects of only IOP and ICP changes to the eye. The perfused system allows us to keep the posterior eye healthy and maintained for long periods of time without the requirement of vascular perfusion. In addition, for this study we wanted to observe the effects of ICP without the impacts of the lymphatic/glymphatic system and how it would impact axonal transport activity.

In addition, due to COVID-19 precautions, we did have a limited sample size of human donor eyes. Given the significant variability of human donor tissue regarding genetics, race, age, and medical history of donors, increasing the sample size would be important to obtain significance. The future availability of donor tissue and scaling of tissue using the newly designed 16-chamber perfusion system (Supplementary Fig. 3e, f) would mitigate this issue. However, incorporating variability in the samples is necessary for ensuring validity of the model, such that broad implications can be made about potential therapeutic options.

To conclude, our study proposes an ex vivo human model for studying SANS that could potentially be used to validate therapies or countermeasures targeting translaminar pressure changes in the eye. With our model we identified morphologic, apoptotic, and inflammatory changes occurring due to the various simulated SANS conditions. Weightlessness has the potential to induce mildly but chronically elevated ICP with an initial increase in IOP during early ascent. This chronic IOP/ICP mismatch between the compartments of the lamina cribrosa can cause anterior movement of the optic disc with visual impairment^22^. Elaborating on these investigations will lead to a better understanding of the pathogenic mechanisms for SANS and allow us to investigate potential treatment strategies associated with IOP and ICP changes during space travel. This allows us to test therapies ex vivo while maintaining the capacity to be translatable to the clinic.

In future studies, we can potentially modify TAS to be placed under zero-gravity chambers or be taken in short-term and long-term flight studies to study the effects of microgravity on human donor posterior eye cups seeded with stem cells. This strategy will provide a platform for future testing with donor stem cells from astronauts and open an avenue for assessment of other factors in space traveler cohorts.

## Methods

### Human subjects

The human samples utilized were de-identified human donor eyes. The methods were performed in accordance with relevant guidelines and regulations and approved by North Texas Health Science Center on Not Human Subject Research (NHSR). It is considered as a NHSR project because it includes “Research accessing only a limited data set or deidentified data set”. The research activity involved de-identified biospecimens obtained from an external vendor (Lions Eye Institute for Transplant and Research) which meets the federal definition of NHSR. Therefore, the samples were not a part of Human Subject Research and did not require Institutional Review Board and Human Subjects Office review of non-human subjects’ research. The human donor eyes utilized for our study were assessed in an unbiased manner using well-established serological testing performed by the Lions Eye Institute. Only eyes having a non-reactive serology were utilized, including negative COVID-19 serologies. We used male (*n* = 10) and female (*n* = 8) donors regardless of gender bias (Supplementary Table 1). All experiments were performed in three independent repeats and relevant analysis performed in a masked manner. Human posterior segment culture experiments from human donors were performed in accordance with University of North Texas Health Science Center Institutional Biosafety Committee approval (proposal # *IBC-2019-0110*). Our manuscript complies with the inclusion and ethics criterion in research.

### Posterior segment perfusion culture

Human donor eyes were harvested within 6–12 h of death and cultured within 24 h in the TAS model^23^. Culture media consisted of a neurobasal perfusion medium, which was prepared fresh prior to receipt of the eyes. The neurobasal perfusion medium was prepared according to previously established protocols^42,43^. Briefly, neurobasal/B27 medium (ThermoFisher Scientific) was utilized with 100 U/ml penicillin (Sigma Aldrich), 100 μg/ml streptomycin (Sigma Aldrich), 1 mM pyruvate (ThermoFisher Scientific), 2 mM glutamine (ThermoFisher Scientific), 5 μg/ml insulin (Sigma Aldrich), 100 μg/ml transferrin (Sigma Aldrich), 100 μg/ml bovine serum albumin (Sigma Aldrich), 60 ng/ml progesterone (Sigma Aldrich), 16 μg/ml putrescine (Sigma), 40 ng/ml sodium selenite (Sigma Aldrich), 40 ng/ml thyroxine (Sigma Aldrich), 40 ng/ml tri-iodothyronine (Sigma Aldrich), 5 μM forskolin (Sigma Aldrich) and 1% human serum (Sigma Aldrich). Under sterile conditions, whole eyes were initially soaked in a povidone-iodine solution for 2 min followed by a wash in sterile phosphate-buffered solution (PBS). Excess adnexa was dissected from the globe, and the eye was bisected at the equator into anterior and posterior segments. Vitreous humor, lens, and the optic nerve sheath were removed from the posterior segment 2 mm from the posterior end of the globe for all samples. Additional sclera was trimmed, if necessary, to ensure a proper fit over the rounded dome of the IOP chamber (bottom compartment) with the ON facing upwards. The chamber dome is slightly flattened at the top which ensures effective perfusion of the retina. The posterior segment was secured with a circular ring sealed using an epoxy resin O-ring with four screws, ensuring a tight seal. Inflow and outflow tubing was inserted into their respective ports, and the posterior eye cup was manually infused with neurobasal perfusion medium using the push/pull method to remove any air bubbles through the outflow port. The ICP chamber (top compartment) was subsequently placed directly on top of the IOP chamber and sealed with four screws, ensuring that the ON was encased within the ICP chamber. Inflow and outflow tubing was inserted and the ICP chamber was infused with neurobasal perfusion medium as described above. The entire TAS unit was incubated at 37 °C in 5% CO_2_ for the duration of the perfusion study. Each chamber was connected to its own hydrostatic pressure transducer attached to a multichannel bridge amplifier. Each pressure transducer was manually calibrated just prior to the study using a two-point liquid calibration method. Medium was perfused through the inlets at an adjustable flow rate using an automated syringe pump system to maintain the desired pressure, and the conditioned medium was collected every 48–72 h from the chamber outlets. Pressure data was recorded every 1 min and averaged over a 24-hour period with the LabChart software system. Flow rates were adjusted manually to maintain the targeted pressures. Basic flow rates ranged from 100 to 300 uL/min to mimic the approximate range of physiologic flow rates^22^. The mean and standard deviations of IOP, ICP, and TLPD were calculated for every 24-hour period. After a 10 to 12-day period, retinal and ON tissue was collected for further analysis. Measurements were taken from distinct samples.

### Imaging posterior segments

To assess changes from the perfusion culture system, images of the posterior globes for each group were recorded pre and post perfusion in the TAS model. Images were captured by an iPhone 11 Pro (Apple Incorporated), and the posterior segment depth and diameter was measured in pixels using Adobe Photoshop. Pixel length was then converted into millimeters for comparative analysis.

### Full field flash ERG recording of ex vivo retinal explants

A retinal tissue sample was collected peripheral to the ONH region and placed in a dish containing neurobasal medium. The samples were placed in a dark chamber for 20 min to dark adapt prior to ERG evaluation. Under darkroom/scotopic conditions, the retinal tissue sample was dissected into two pieces. Each retinal segment was carefully centered onto the OcuScience ex vivo sample holder with one piece flipped to ensure that one of the retinal segments was oriented correctly with retinal photoreceptors facing upwards. The OcuScience ERG system was set up according to the manufacturer’s specifications. Temperature (37 °C), impedance (≤20 KOhms), and offset voltage (≤10 mV) were adjusted in preparation for presentation of the stimulus. If resistance was too high, any residual air bubbles were removed from the capillary tubing. Tissue samples were perfused with AMES solution at 50 ml/hr, and full field flash ERG recordings were measured with the handheld multi-species ERG unit (HMsERG). The light stimuli impulse was generated in a Ganzfeld dome with light emitting diodes. The ERG responses were recorded by stimulating the retina with a flash intensity which included sequential phases of 0.03 cd pulse (10 sweeps, 60 s between each sweep), 0.1 cd pulse (10 sweeps, 60 s between each sweep), 0.3 cd pulse (10 sweeps, 60 s between each sweep), 1.0 cd pulse (10 sweeps, 60 s between each sweep) and 3.0 cd pulse (10 sweeps, 60 s between each sweep). All intervals were averaged. The amplitudes were measured from the baseline to the positive peak of each waveform and latency measured by time-to-peak major positive deflection. Data were analyzed amongst all groups.

### Cholera Toxin B assay

Cholera toxin B (CTB) (Catalog number # C-34778, ThermoFisher Scientific) was used to measure the retrograde transport activity through the ONH. CTB conjugated to Alexa Fluor 647 was resuspended to 1 mg/ml in 0.100 ml of PBS. The solution was perfused through the IOP chamber for a 48-hour duration before harvest. Tissue was collected from the ONH and fixed in formalin. The tissue was paraffin embedded and sectioned in sagittal planes at 5 μm for IHC. Fluorescent images were captured using a Nikon Eclipse Ti2 inverted microscope (Nikon, Melville, NY). Quantification was performed by a masked observer through Adobe Photoshop count tool and graphed using GraphPad Prism 6.

### Western immunoblot analysis

For western immunoblot analysis, conditioned perfusion medium was collected. Concentrating beads (StrataClean Resin, Agilent) were added to the conditioned medium and centrifuged, after which the supernatant was discarded. An equal volume of 2X Laemmli buffer was added, boiled, and separated by SDS-PAGE. Protein was transferred to nitrocellulose membranes and blocked with SuperBlock T20 (Thermo Fisher Scientific). Blots for the conditioned medium were incubated with one of the following primary antibodies overnight at room temperature: rabbit anti-COLIV (1:1000, NB120-6586; Novus), and mouse anti-FN (0.5–1 ug/ml, AB200541; Abcam). The blots were then incubated for 1 h at room temperature with HRP-linked anti-mouse or anti-rabbit secondary antibodies (goat anti-rabbit, SC-2004, 1:1,000, goat anti-mouse, SC-2005, 1:1,000). Chemiluminescent signal was developed with SuperSignal West Femto Substrate (Thermo Fisher Scientific) and images were captured with the ChemiDoc MP Imaging System (Bio-Rad, Hercules, CA). Original blots depicted in Fig. 4 have been displayed as uncropped blots in Supplementary Fig. 2. All blots derive from the same experiment and were processed in parallel.

### TaqMan® human retina assay

For the retinal tissue sample, approximately 50% of the peripheral retina was collected and lysed with cell lysis buffer (M-PER™ Mammalian Protein Extraction Reagent, ThermoFisher), β-mercaptoethanol, and a steel ball. The retina was then homogenized with TissueLyser LT (Qiagen) for 10 min at 50 oscillations/sec. RNA and protein was extracted with the NucleoSpin RNA/Protein Mini kit (Macherey-Nagel) (M-PER lysis buffer with protease/phosphatase cocktail was substituted for PSB-TCEP). The RNA was utilized for the TaqMan assay. After RNA extraction from the retinal samples with the NucleoSpin RNA/Protein Mini kit (Macherey-Nagel), RNA concentration was measured using the NanoDrop 2000c (Thermo Fisher Scientific). 2.5 ng of RNA was reverse transcribed to cDNA (iScript Reverse; Bio-Rad Labs Inc., Richmond, CA, USA) with RT-PCR using the QTC-100 Thermal Control Thermocycler (BioRad Hercules, CA). The parameters were 25 °C for 5 min, 46 °C for 20 min, 95 °C for 1 min, and 4 °C for 24 h. Custom TaqMan Gene Expression Array Plate 0.1 mL (Fast) were utilized for the study. Expression levels for genes of interest were determined with hydrolysis probes (FAM dye labeled) commercially available as ThermoFisher Scientific probes with optimized primer and probe concentrations embedded on the plates (Supplemental Table 2). The expression of 22 genes of interest and four selected reference genes (18 s rRNA, GAPDH, HPRT, GUSB) was examined by real-time TaqMan® PCR assay. The plates were prepared with 260 uL of cDNA for each sample (*N* = 6) according to the manufacturer’s specifications. Quantification was accomplished on a QuantStudio3 (Applied Biosystems) using TaqMan® Universal PCR Master Mix and universal thermocycling parameters of 50 °C for 2 h and 95 °C for 20 min. The RT-PCR samples were run in duplicates using 1 μL cDNA. For the RNA analysis, the samples were compared to the internal GAPDH expression values to calculate the ΔCq values and log2 fold values calculated for all the groups (G1-G6). The fold values were based on *p* < 0.05. Graphical representation was performed by GraphPad Prism 6.

### Paraphenylenediamine stain of optic nerves

A portion of the ON was processed for paraphenylenediamine (PPD) staining. After perfusion, a suture was placed at the distal end of the ON to orient the tissue. The ON was removed and fixed in a 2% paraformaldehyde/2.5% glutaraldehyde mixture in 0.1 M sodium cacodylate buffer. After fixation, the ON samples were rinsed with 0.1 M sodium cacodylate and incubated overnight with 2% osmium. The ON samples were washed with 0.1 M sodium cacodylate and dehydrated before embedding in BEEM capsules (Electron Microscopy Services). Cross sections of the ON were cut at 0.75–1.0 μm, and carefully placed on a drop of water and heated to facilitate adherence to the slide. The slides were then stained with 1% p-phenylenediamine in isopropanol: methanol (1:10) for 10–30 min and subsequently washed with isopropanol. Double masked manual axon counts of normal and abnormal axons were done for each sample.

### Hematoxylin and eosin staining and immunohistochemistry (IHC)

Following collection of retinal and ONH tissue samples for analysis, the remaining posterior segment was fixed in formalin and paraffin embedded. The posterior segments were sectioned in sagittal tissue planes at 5μm. H&E processing and staining was performed within the histology core. For IHC, the paraffin-embedded segments were processed in an automated staining system for deparaffinization using a 100% xylene, 95% ethanol, and 50% ethanol solution and subsequently rehydrated with PBS. Following incubation for 1 h at room temperature with SuperBlock T20 (Thermo Fisher Scientific), the sections were incubated overnight at room temperature with one of the primary antibodies: rabbit anti-LAM (1:100, NB-300-144; Novus Biologicals), rabbit anti-FN (1:100, AB 1954; Millipore), rabbit anti-COLIV (1:100, NB-120-6586; Novus Biologicals), rabbit anti-TLR4 (1:50, AB13556; Abcam), mouse anti-TNFα (1:50, AB22048; Abcam) and mouse anti-GFAP (0.2–2 ug/ml, MA5-12023; Invitrogen). The sections were then incubated for 1–2 h at room temperature with Alexa Fluor secondary antibodies (Alexa Fluor 488 goat anti-rabbit, A11008, 1:500 and Alexa Fluor 594 donkey anti-mouse, A21203, 1:500). Cell nuclei were counterstained with DAPI/Antifade solution (ThermoFisher). Fluorescent IHC mages and brightfield H&E images were captured using a Nikon Eclipse Ti2 inverted microscope (Nikon, Melville, NY). Grading scale with a scale of none, low, medium, and high was performed by a double masked observer and results graphed using GraphPad Prism 6.

### TUNEL assay

To perform TUNEL stain, the paraffin-embedded segments were processed in a manual system for deparaffinization using a 100% xylene, 95% ethanol, and 50% ethanol solution and subsequently rehydrated with PBS. Following incubation for 1 h at room temperature with SuperBlock T20 (Thermo Fisher Scientific), the sections were stained according to manufacturer’s protocol for the TUNEL assay (catalog number ab66108, Abcam). The detection kit utilizes terminal deoxynucleotidyl transferase (TdT) to catalyze incorporation of fluorescein-12-dUTP at the free 3’-hydroxyl ends of the fragmented DNA. The fluorescein-labeled DNA was observed by fluorescence microscopy. Cell nuclei were counterstained with DAPI/Antifade solution (ThermoFisher). Fluorescent IHC mages and brightfield Images were captured using a Nikon Eclipse Ti2 inverted microscope (Nikon, Melville, NY). Quantification was performed by a masked observer through Adobe Photoshop count tool and graphed using GraphPad Prism 6.

### Statistical analysis

One and two-way analysis of variance was used to compare samples of different treatment groups and intergroup differences between time points. Measurements were taken from distinct samples. GraphPad Prism 6 (San Diego, CA) was used to perform all statistical analyses. Data are presented as mean ± standard error of the mean and *P* < 0.05 was considered statistically significant.

### Reporting summary

Further information on research design is available in the Nature Research Reporting Summary linked to this article.

## Supplementary information

Supplemental Data

Reporting Summary Checklist

## Data Availability

The datasets generated during and/or analyzed during the current study are available from the corresponding author on reasonable request.
