# Supplementary material for: The ex vivo human translaminar autonomous system to study spaceflight associated neuro-ocular syndrome pathogenesis
Source: NPJ Microgravity. 2022 Oct 28;8:44. doi: 10.1038/s41526-022-00232-5 (PMC9616431; doi:10.1038/s41526-022-00232-5)
Supplement: Supplementary file 1 — Supplemental Data [file 41526_2022_232_MOESM1_ESM.pdf]

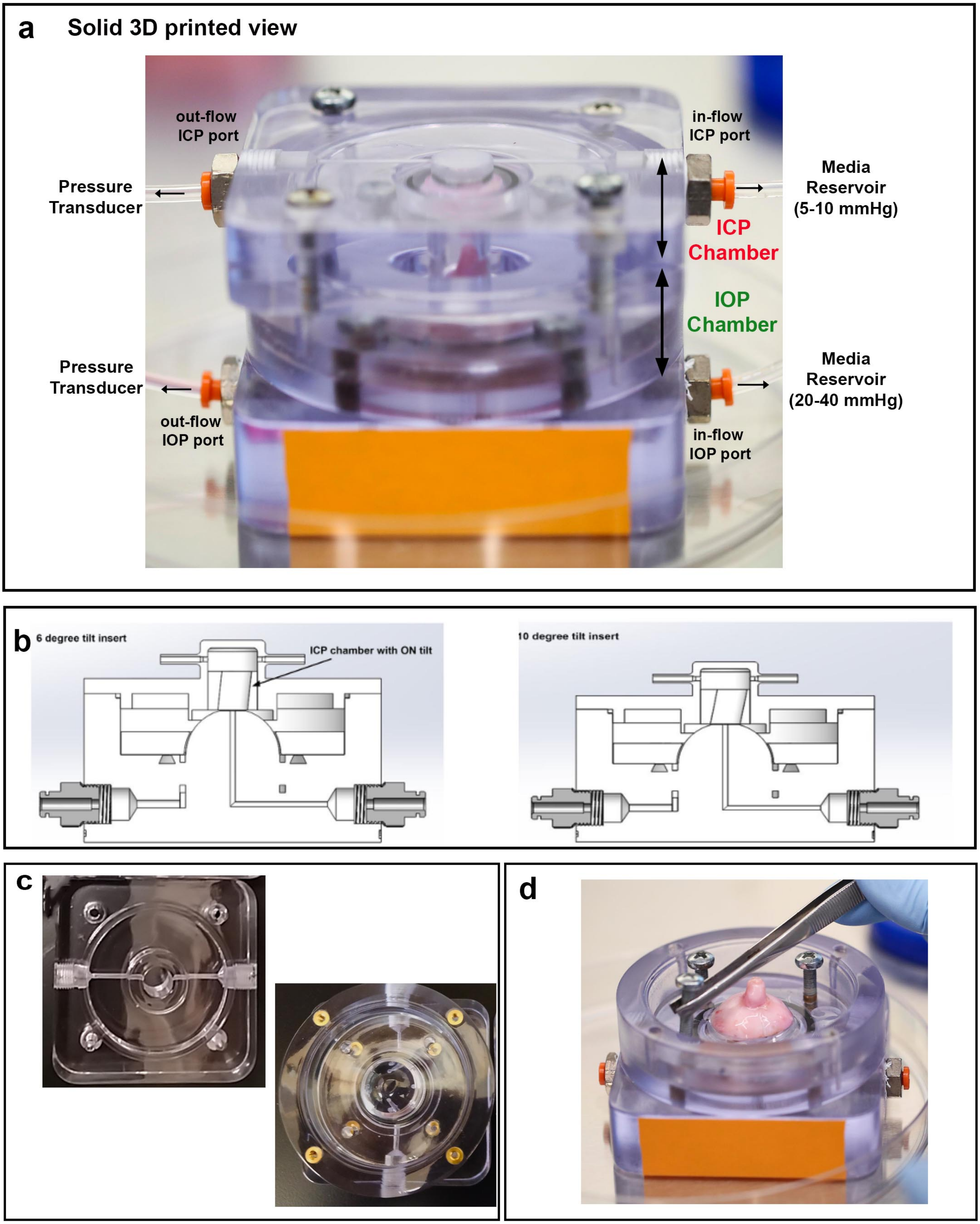

Supplemental Figure 1: Translaminar autonomous system Model depiction. (a) Solid 3D printed view. with chambers depicted (b) Diagrammatic view of 6° tilt and 10° tilt. Actual 3D printed stereolithography (Clearvue Polished) model (c) top chamber lid and bottom chamber with (d) a human posterior segment depicted in the model. All images are copyright of Tasneem Sharma, 2022. All rights reserved

a. Fibronectin

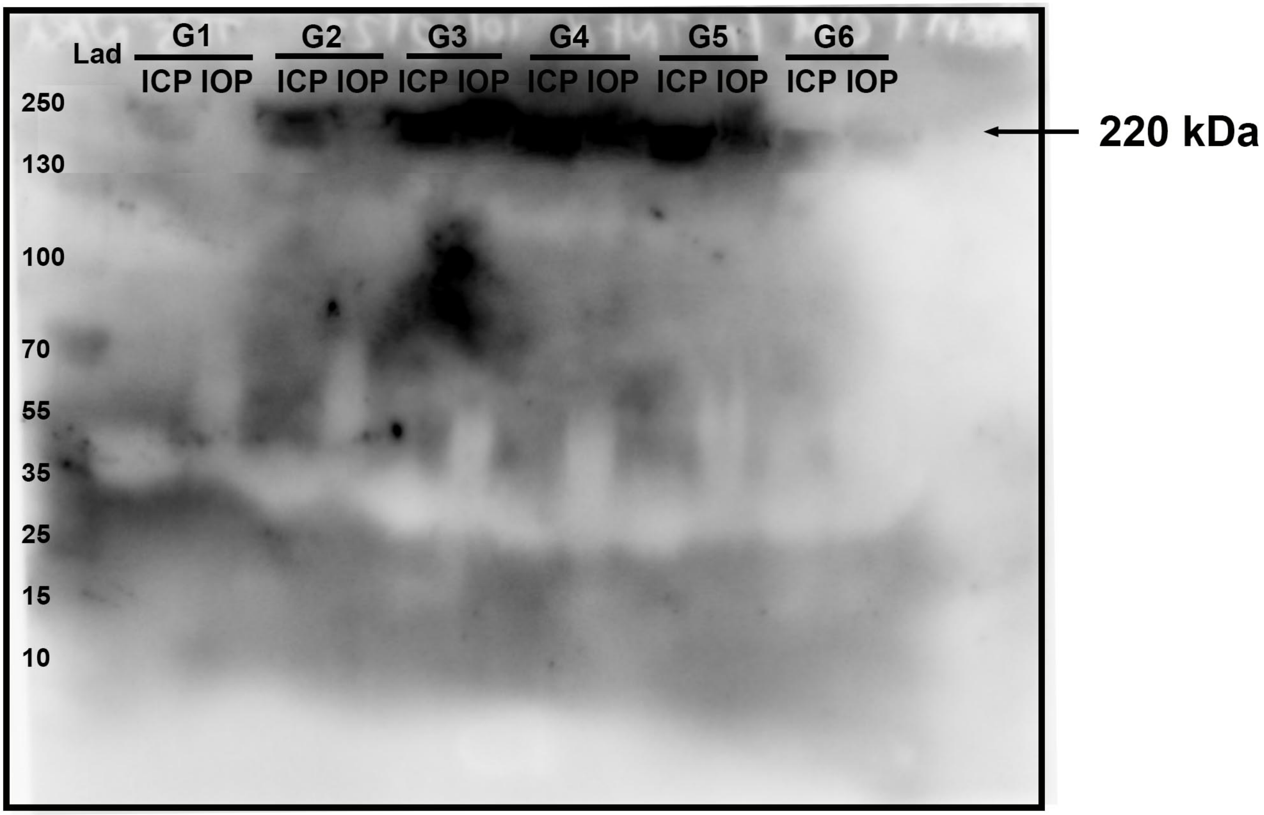

b. Collagen IV

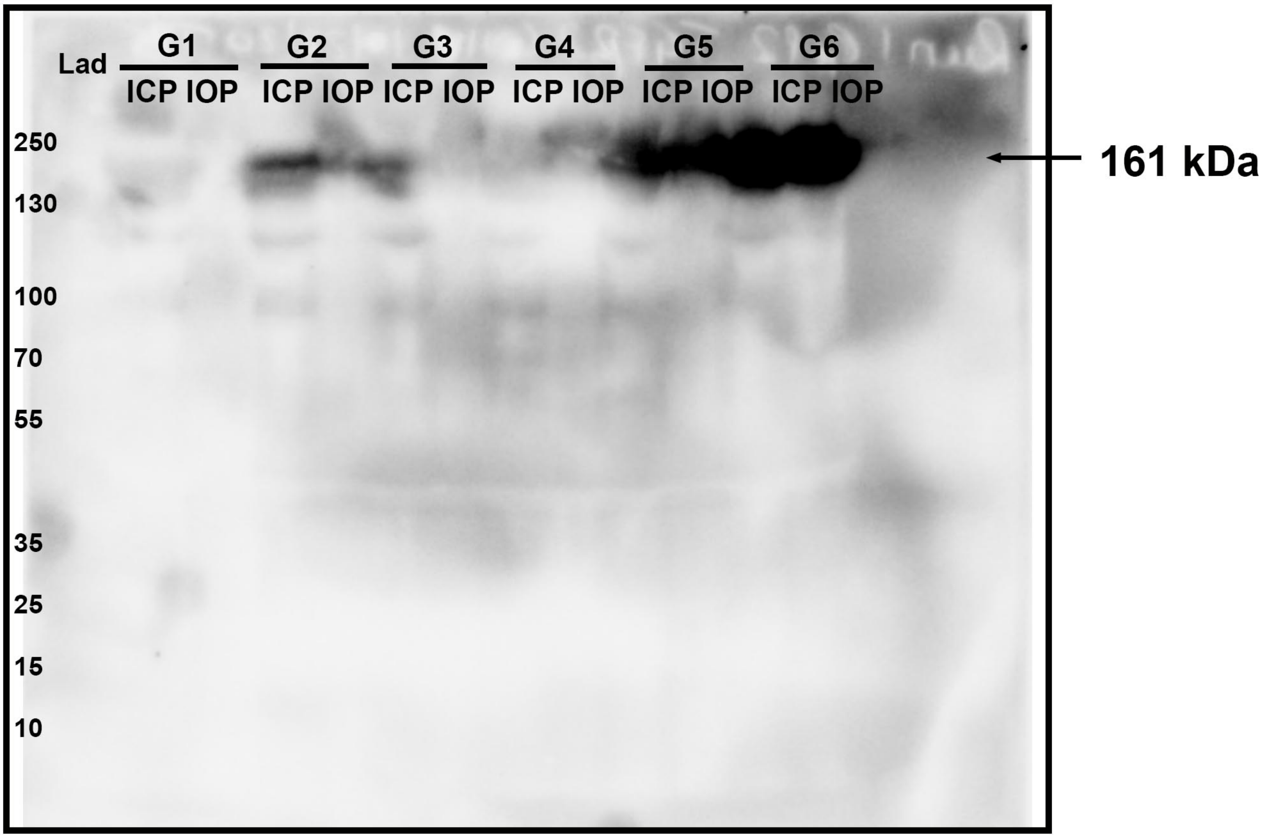

Supplemental Fig. 2: Western blots of conditioned medium collected from the IOP and ICP chambers of every group throughout 14-day perfusion culture Densitometric values of (a) FN and (b) COLIV. ICP=intracranial pressure; IOP=intraocular pressure; G1 = group 1; G2 = group 2; G3 = group 3; G4 = group 4; G5 = group 5; G6 = group 6; N=3. All images are copyright of Tasneem Sharma, 2022. All rights reserved

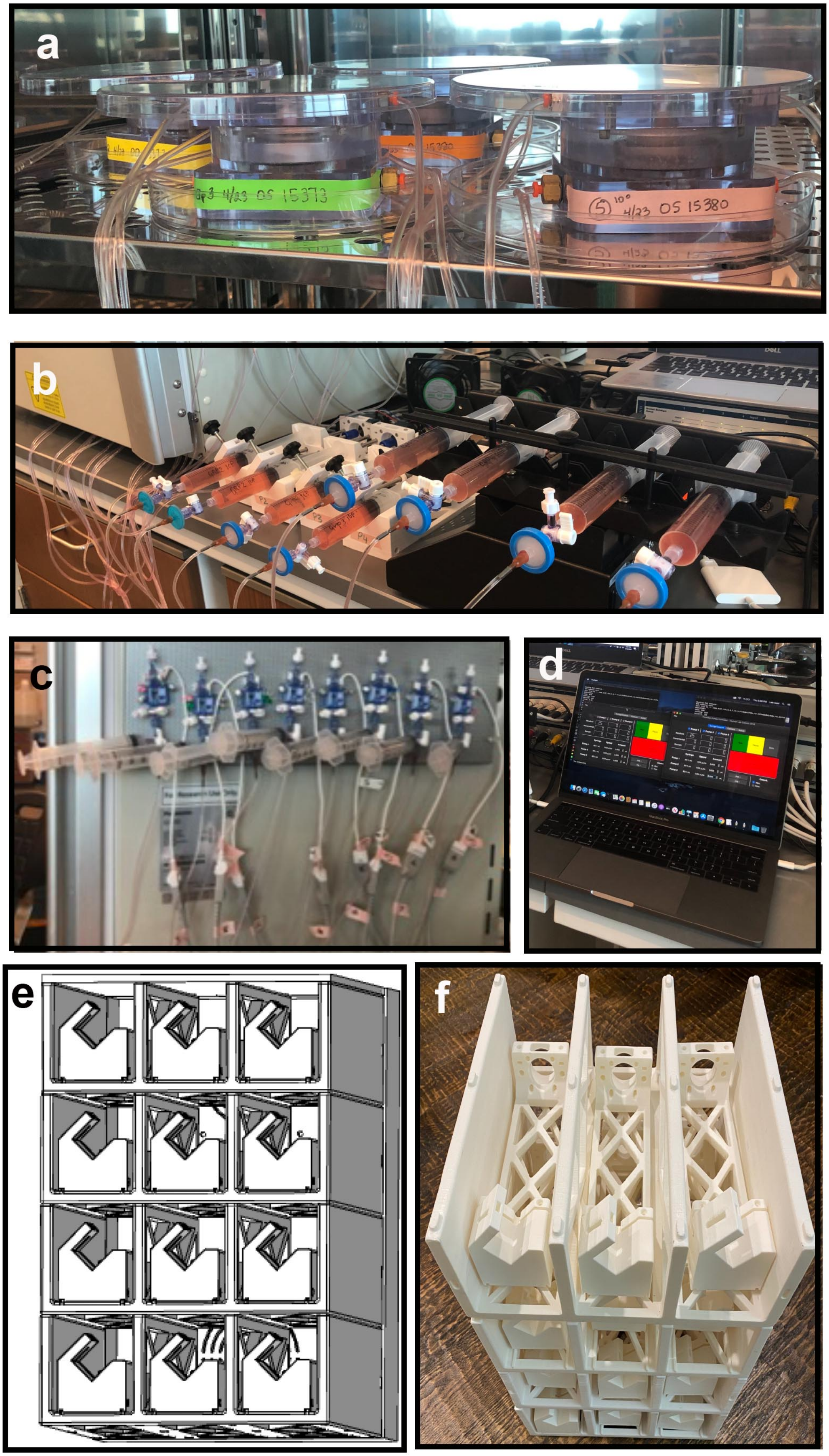

Supplemental Fig. 3: Mechanics of the translaminar autonomous system and newly modeled 16 chamber system. Experimental setup for the TAS model. (a) Image of the TAS model with all the fittings in place and (b) representation of inflow and outflow reservoir syringes on individualized pumps. (c) Perfusion of medium through an infusion pump with an exit empty syringe on the transducer end to collect conditioned medium in (d) an automated fashion through computational modeling. Depiction of the newly designed 16-chamber perfusion system with (e) solid front view and (f) actual 3D printed stereolithography (Clearvue Polished) model. All images are copyright of Tasneem Sharma, 2022.
All rights reserved

Supplemental Table 1: Tabulation of donor information. Tabular information of human donors utilized within the study with identification of gender, age, race, and diagnosis

Subject#	Eye	Gender	Age	Race	Group	Days on System	Diagnosis
7346	OD	Female	64	Caucasian	1	14	Myocardial infarction, Lung cancer (dx 2019, active), Mets to liver (active), Thyroid cancer
7346	OS	Female	64	Caucasian	2	14	
15373	OD	Female	86	Caucasian	3	14	Pancreatic cancer with mets to kidneys. HTN, Hyperthyroidism, Shingles, Gout, Recurrent
15373	OS	Female	86	Caucasian	4	14	
15380	OD	Female	89	Caucasian	5	14	Bowel obstruction. Interstitial lung disease, HTN, GERD, PVD, Hypothyroidism, Ulcerative
15380	OS	Female	89	Caucasian	6	14	
18004	OD	Male	82	Caucasian	1	13	Hip Fracture, Stroke, AFib, COPD, HTN, Kidney disease, Stroke, COVID test negative
18004	OS	Male	82	Caucasian	2	13	
20779	OD	Male	78	Caucasian	3	12	Pancreatic cancer, Weakness, Hypokalemia
20779	OS	Male	78	Caucasian	4	12	
22196	OD	Female	55	Caucasian	5	12	None Listed
22196	OS	Female	55	Caucasian	6	12	
22536	OD	Male	77	Caucasian	1	12	Leukemia, thrombocytopenia, transfusions (O-neg 6/14 & 6/15, platelets 6/15)
22536	OS	Male	77	Caucasian	2	12	
32115	OD	Male	77	Caucasian	3	12	Brain CA, PMH: Afib, COPD, Smoker, brain CA
32115	OS	Male	77	Caucasian	4	12	
34085	OD	Male	87	Caucasian	5	14	ESLD, Abdominal pain 2/2 TURP on 8/14. COVID NEG 8/18.
34085	OS	Male	87	Caucasian	6	14	

Supplemental Table 2: Tabulation of custom TaqMan gene expression array plate probes embedded on the plates

ThermoFisher Scientific Assay ID	Gene Symbol(s)	Gene Name(s)
Hs99999901 sl	18s rRNA	Eukaryotic 18S ribosomal RNA
Hs99999905 ml	GAPDH	Glyceraldehyde-3-Phosphate Dehydrogenase
Hs99999909 ml	HPRT	Hypoxanthine Phosphoribosyltransferase 1
Hs99999908 ml	GUSB	Glucuronidase Beta
Hs02758991 gl	GAPDH	glyceraldehyde-3-phosphate dehydrogenase
Hs01048932 gl	BCL2	BCL2, apoptosis regulator
Hs00180269 ml	BAX	BCL2 associated X, apoptosis regulator
Hs00234387 ml	CASP3	caspase 3
Hs00169152 ml	CASP7	caspase 7
Hs01018151 ml	CASP8	caspase 8
Hs00152939_ ml	TLR4	toll like receptor 4
Hs00174128 ml	TNF	tumor necrosis factor
Hs00909233 ml	GFAP	glial fibrillary acidic protein
Hs00609286 ml	GSS	glutathione synthetase
Hs00610419_gl	AIFI	allograft inflammatory factor 1
Hs01060992 ml	RBPMS	RNA binding protein with multiple splicing
Hs00366711 ml	POU4FI	POU class 4 homeobox 1
Hs00231820 ml	POU4F2	POU class 4 homeobox 2
Hs00231275_ ml	POU4F3	POU class 4 homeobox 3
Hs00158126 ml	ISLI	ISL LIM homeobox 1
Hs06633377 sl	THYI	Thy-1 cell surface antigen
Hs00606024 ml	NEFH	neurofilament heavy polypeptide
Hs00196245_ ml	NEFL	neurofilament, light polypeptide
Hs00892431 ml	RHO	rhodopsin
Hs00925200 ml	PRKCA	protein kinase C alpha
Hs00270282 ml	STXIA	syntaxin lA
